# Plant Evolution History Overwhelms Current Environment Gradients in Affecting Leaf Chlorophyll Across the Tibetan Plateau

**DOI:** 10.3389/fpls.2022.941983

**Published:** 2022-07-11

**Authors:** Yicheng He, Tingting Li, Ruiyang Zhang, Jinsong Wang, Juntao Zhu, Yang Li, Xinli Chen, Junxiao Pan, Ying Shen, Furong Wang, Jingwen Li, Dashuan Tian

**Affiliations:** ^1^Key Laboratory of Ecosystem Network Observation and Modeling, Institute of Geographic Sciences and Natural Resources Research, Chinese Academy of Sciences (CAS), Beijing, China; ^2^School of Ecology and Nature Conservation, Beijing Forestry University, Beijing, China; ^3^Faculty of Natural Resources Management, Lakehead University, Thunder Bay, ON, Canada; ^4^Key Laboratory of Animal Ecology and Conservation Biology, China Institute of Zoology, Chinese Academy of Sciences (CAS), Beijing, China; ^5^College of Resources and Environment, University of Chinese Academy of Sciences, Beijing, China

**Keywords:** alpine grassland, evolutionary history, leaf chlorophyll, photosynthesis, photosynthetically active radiation, plant functional group

## Abstract

**Aims:**

Leaf chlorophyll (Chl) is a fundamental component and good proxy for plant photosynthesis. However, we know little about the large-scale patterns of leaf Chl and the relative roles of current environment changes vs. plant evolution in driving leaf Chl variations.

**Locations:**

The east to west grassland transect of the Tibetan Plateau.

**Methods:**

We performed a grassland transect over 1,600 km across the Tibetan Plateau, measuring leaf Chl among 677 site-species.

**Results:**

Leaf Chl showed a significantly spatial pattern across the grasslands in the Tibetan Plateau, decreasing with latitude but increasing with longitude. Along with environmental gradient, leaf Chl decreased with photosynthetically active radiation (PAR), but increased with water availability and soil nitrogen availability. Furthermore, leaf Chl also showed significant differences among functional groups (C_4_ > C_3_ species; legumes < non-legume species), but no difference between annual and perennial species. However, we surprisingly found that plant evolution played a dominant role in shaping leaf Chl variations when comparing the sum and individual effects of all the environmental factors above. Moreover, we revealed that leaf Chl non-linearly decreased with plant evolutionary divergence time. This well-matches the non-linearly increasing trend in PAR or decreasing trend in temperature during the geological time-scale uplift of the Tibetan Plateau.

**Main Conclusion:**

This study highlights the dominant role of plant evolution in determining leaf Chl variations across the Tibetan Plateau. Given the fundamental role of Chl for photosynthesis, these results provide new insights into reconsidering photosynthesis capacity in alpine plants and the carbon cycle in an evolutionary view.

## Introduction

Leaf chlorophyll (Chl) is fundamental for harvesting light for plant photosynthesis (Croft et al., 2017). The importance of leaf Chl for photosynthesis has been comprehensively evaluated at both ecosystem and species levels. Across terrestrial ecosystems, numerous studies, using remote sensing techniques, reveal the close relationships between leaf Chl fluorescence and photosynthetic processes, such as rubisco concentration, maximum rate of carboxylation (*V*cmax), and electron transport (*J*max) (Carswell et al., 2000; Kattge et al., 2009; Luo et al., 2019). At the species level, leaf Chl has been mostly studied at the local scale (Croft et al., 2017). However, across contrasting environments, species-level Chl might show great variations due to the difference in plant evolutionary histories, climate, soil, and functional groups (Li et al., 2018a,b; Zhang et al., 2020). To date, we still know little about the large-scale patterns and key drivers of species-level leaf Chl variations (Ryu et al., 2019).

Plant evolutionary history may be crucial to drive leaf Chl variations (Schmerler et al., 2012). Previously, leaf Chl is found to show evolutionary divergence among 823 woody species across forest ecosystems (Li et al., 2018a), denoting the potential role of evolutionary history in affecting leaf Chl. However, we do not know the direction of evolutionary effects on leaf Chl (Li et al., 2018a). Coincidently, the geological time-scale uplift of the Tibetan Plateau keeps pace with plant evolutionary history, providing an ideal opportunity to test how plant evolution regulates the direction of leaf Chl variations. Specifically, following the collision of the India and Eurasia continent (*c*. 50 million years ago), the Tibetan Plateau begins to uplift rapidly (Royden et al., 2008; Wang et al., 2008). This should cause directional changes in environmental conditions, especially for photosynthetically active radiation (PAR) and temperature. Based on the well-known correlations of PAR or temperature with altitude, PAR should gradually increase but temperature decreases during the uplift (Blumthaler et al., 1997). These environmental changes during plant evolutionary history may decrease the leaf Chl in newly evolved species across the Tibetan Plateau, due to the evolutionary adaptation to extremely high PAR (avoiding radiation damage) and cold environment (more resource allocation to plant survival) (Knapp and Smith, 2001).

In addition to plant evolution, current climate and soil nutrients might affect leaf Chl as well (Anjum et al., 2011; Genesio et al., 2020). Generally, plants tend to enhance leaf Chl in light-limited environments, helping to compete for the limiting light resource and sustain plant growth (Hallik et al., 2009; Li et al., 2018a; Genesio et al., 2020). For example, leaf Chl is found to be higher for trees' understory than in the canopy (Niinemets, 2010). Low precipitation normally causes soil water stress and air dryness, negatively influencing the elasticity of chloroplast membrane and the synthesis of leaf Chl (Rastogi et al., 2020; Xiong and Nadal, 2020). Soil nutrient availability may influence leaf Chl *via* altering plant nutrient uptake and use. For instance, leaf Chl is generally stimulated by more nitrogen (N) input (Cai et al., 2011; Guo et al., 2014; Rhein and Silva, 2016), because N is the most essential but limiting element to synthesize leaf Chl (Cai et al., 2011; Croft et al., 2017). Phosphorus (P) is also the basic element for plant photosynthesis, such as adenosine triphosphate (ATP), phosphodiester linkage (Lambers et al., 2011), and 5-aminolevulinic acid (ALA, a basic compound for Chl synthesis) (Masuda et al., 1996). Based on the importance of P for energy biosynthesis (ATP) and ALA, leaf Chl may vary with soil P availability.

Plant functional group (PFG) has the potential to determine leaf Chl variations (Zhang et al., 2020; Kelly et al., 2021). For example, C_4_ plants have the ability to increase intercellular CO_2_ concentration, which leads to a stronger photosynthetic capacity than C_3_ plants (Wang et al., 2011; Osborne et al., 2014). It is thus expected that C_4_ species might have a higher leaf Chl to utilize the higher CO_2_ concentration for maximizing photosynthesis. Among PFG with different life spans, annual plants tend to allocate more resources to aboveground growth, while perennial plants invest more in belowground growth to maintain the advantage in competing for soil resources in the long term (Onoda et al., 2017; Monroe et al., 2019). This suggests that annual species adopt a resource-use strategy, but perennial species with a resource-conservative strategy (Wright et al., 2004). This strategy difference likely results in a higher leaf Chl in annual than perennial species, which enables to increase the photosynthetic capacity of annual species for its fast growth. Legume species generally have advantages in obtaining soil N, possibly leading to higher leaf N and Chl than non-legume species (Guo et al., 2017; Zhang et al., 2020). Consequently, leaf Chl may be different among PFGs, due to the difference in the photosynthetic pathway, life span, and nitrogen-use strategy. In spite of these potential mechanisms above, we still do not know the relative importance of plant evolutionary history vs. current environment changes in shaping the patterns of leaf Chl across a range of contrasting environments.

Based on the reasoning above, the uplift of the Tibetan Plateau provides an ideal opportunity to explore the role of plant evolution in shaping leaf Chl variations (Xu et al., 2010; Chen et al., 2013). Furthermore, recent global changes (e.g., warming, precipitation change, and N deposition) have substantially impacted leaf Chl and photosynthetic processes across this area (Fu et al., 2014; Fu and Shen, 2016). Thus, we conducted a grassland transect survey (97 sites) of leaf Chl among common species (93 species from 27 families) and related variables across the Tibetan Plateau. Specifically, we addressed the following questions: (1) What are the regional patterns and key environmental control of leaf Chl concentration among alpine herbaceous species? (2) What is the role of plant evolution in affecting leaf Chl concentration relative to current environmental changes?

## Materials and Methods

### Site Description

We conducted a grassland transect survey of leaf Chl from the east to west of the Tibetan Plateau, covering a large gradient (1,600 km) of climate, soil nutrients, and ecosystem type. In total, we investigated 97 sites (Supplementary Figure 1), spanning a latitude range from 30.24 to 33.67°N, longitude from 80.36 to 92.13°E, and elevation from 4,181 to 5,016 m. The average annual PAR (1981–2010) is between 2461.8 and 2860.4 mol m^−2^, annual temperature between −5.37 and 0.99°C, and annual precipitation between 61 and 441 mm. Ecosystem types included alpine steppe, alpine meadow, and alpine desert grassland. Soil available N varied from 6.48 to 443.71 mg kg^−1^ and available P from 0.94 to 7.11 mg kg^−1^. Detailed geographical information of the sampling sites is presented in Supplementary Table 1.

### Field Survey and Measurement

The field survey was carried out from July to August during the growing season in 2019 and 2020. According to local vegetation, in each site, three 5 m^*^5 m plots were randomly established in a 10 m^*^200 m area. In each sampling site, we randomly selected at least three plant individuals (3~6 replications) for each of the common species (above 80% of the local species) across three plots. Specifically, healthy leaf Chl was measured by the SPAD-502 Chl meter (Minolta, Osaka, Japan) (Uddling et al., 2007; Yuan et al., 2016). We measured the value of SPAD in the middle of the leaf, and leaf veins were excluded in each measurement. To more accurately estimate leaf Chl, the value was obtained by the mean of at least three readings in different positions of each individual. Finally, the mean value of leaf Chl in each site species was estimated at least by three plant individuals. In total, we measured 2,031 individuals among 93 herbaceous species from 27 families (many species occurring in multiple sites). All the species are grouped into legumes (10) vs. non-legume species (83), C_4_ (12) vs. C_3_ species (81), or annual (20) vs. perennial species (68).

Accordingly, three soil samples were randomly collected by a 7.5 cm-diameter soil auger at 0–10 cm depth in each plot. Then, we mixed three soil samples into one composite sample in each plot. The composite soil samples were screened by a 2-mm mesh to remove roots and stones (Pan et al., 2021). Totally, there were three composite soil samples at each site. A part of the soil was in cold storage by the portable refrigerator, and the other part was air-dried in labs. We measured soil available N (AN) by the alkali solution diffusion absorption method. Soil available P (AP) concentration was firstly extracted by the NaHCO_3_ solution (0.5 M) and then analyzed using the UV-visible spectrophotometer (Olsen, 1954).

### Statistical Analyses

Before analyses, we obtained the data for mean annual precipitation (MAP) and temperature (MAT) from the Worldclim Database (http://www.worldclim.org), based on our location information. We then calculated humidity index (HI) as follows: HI = MAP / (MAT +10) (Martonne, 1926). PAR data were acquired from the dataset of “Global radiation, photosynthetically active radiation, and the diffuse components in China, 1981–2010” (Ren et al., 2018).

To obtain the divergence time of our sampling species, we first confirmed whether all the species (93 species from 25 families) have been included by *The Plant List* (http://www.theplantlist.org). Then, we established the phylogenetic tree using the “phytools” and “S.PhyloMaker” packages (Qian and Jin, 2016). The divergence time of each species was quantified by the branch length of each species in the phylogenetic tree (Qian and Jin, 2016). At last, we tested the strength of the phylogenetic signal for leaf Chl, using Blomberg's K and Pagel'λ K analyses with the “Picante” package (Blomberg et al., 2003).

Based on the data above, we first used the *t*-test analysis to compare the difference in leaf Chl between C_3_ and C_4_ species, annuals and perennial species, or legumes vs. non-legume species. Then, the linear or non-linear regression analyses were applied to analyze the pairwise relationships of leaf Chl with plant evolutionary divergence time (family and genus levels), climate (PAR and HI), soil nutrients (AN and AP), and location information (latitude, longitude, and altitude). All the regression analyses were performed by the “stats” package (Flynt and Dean, 2016).

Moreover, we performed the Structural Equation Model (SEM) to distinguish the direct and indirect effects of environmental factors and plant evolution on leaf Chl. Before the SEM analysis, we firstly established a conceptual model of potential casual pathways in driving leaf Chl, based on the traditional knowledge and the correlations between environmental factors (Supplementary Figure 2). Then, the model performance was assessed by the chi-square test (χ^2^ = 10.178, *p* = 0.12) with the “Lavaan” package (Rosseel, 2012). Finally, we analyzed and compared the standardized pathway coefficients of plant evolution and environmental factors on leaf Chl by summing all the direct and indirect effects. All statistical analysis was carried out in *R* software (version 3.6.3, *R* Foundation for Statistical Computing) and all the figures were made using the “ggplot2” package (Ginestet, 2011).

## Results

### The Regional Patterns and Environmental Controls of Leaf Chl

**Figure 1:**
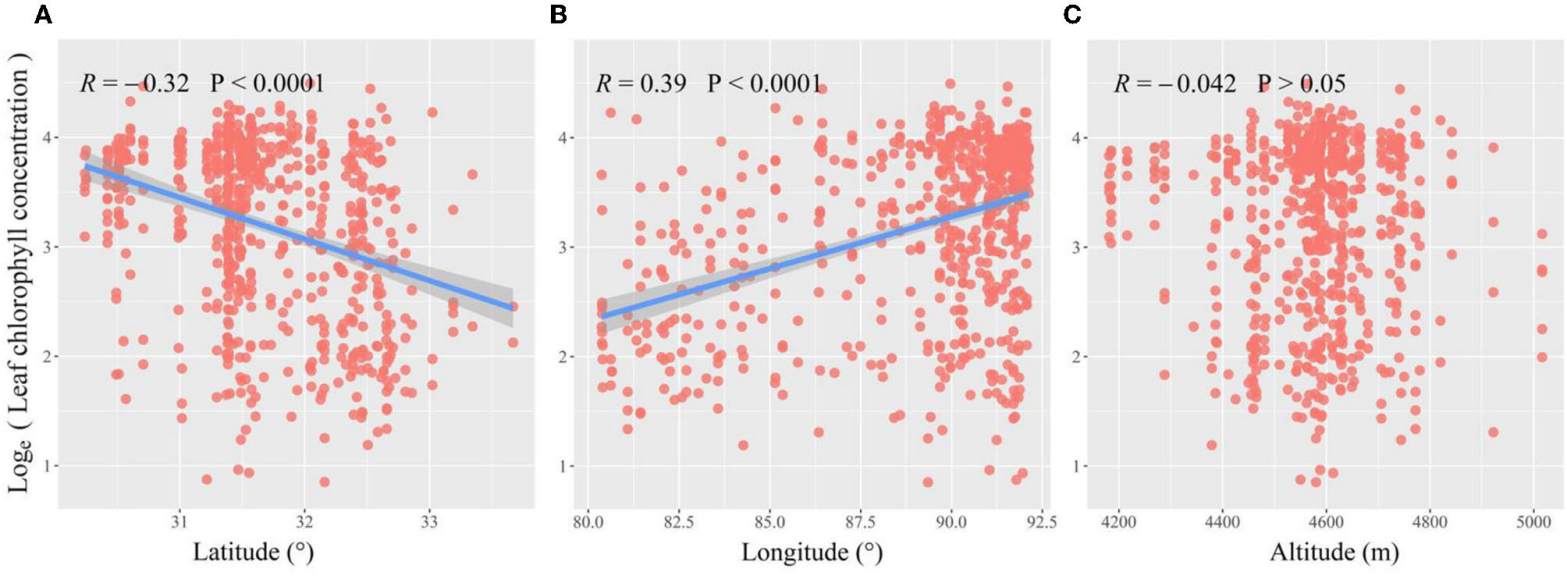
The latitudinal **(A)**, longitudinal **(B)**, or elevation **(C)** patterns of leaf chlorophyll (Chl) across grasslands in the Tibetan Plateau. Red dots represent species-specific observations in each site. The blue lines and shades represent the regression lines with a 95% confidence band.

**Figure 2:**
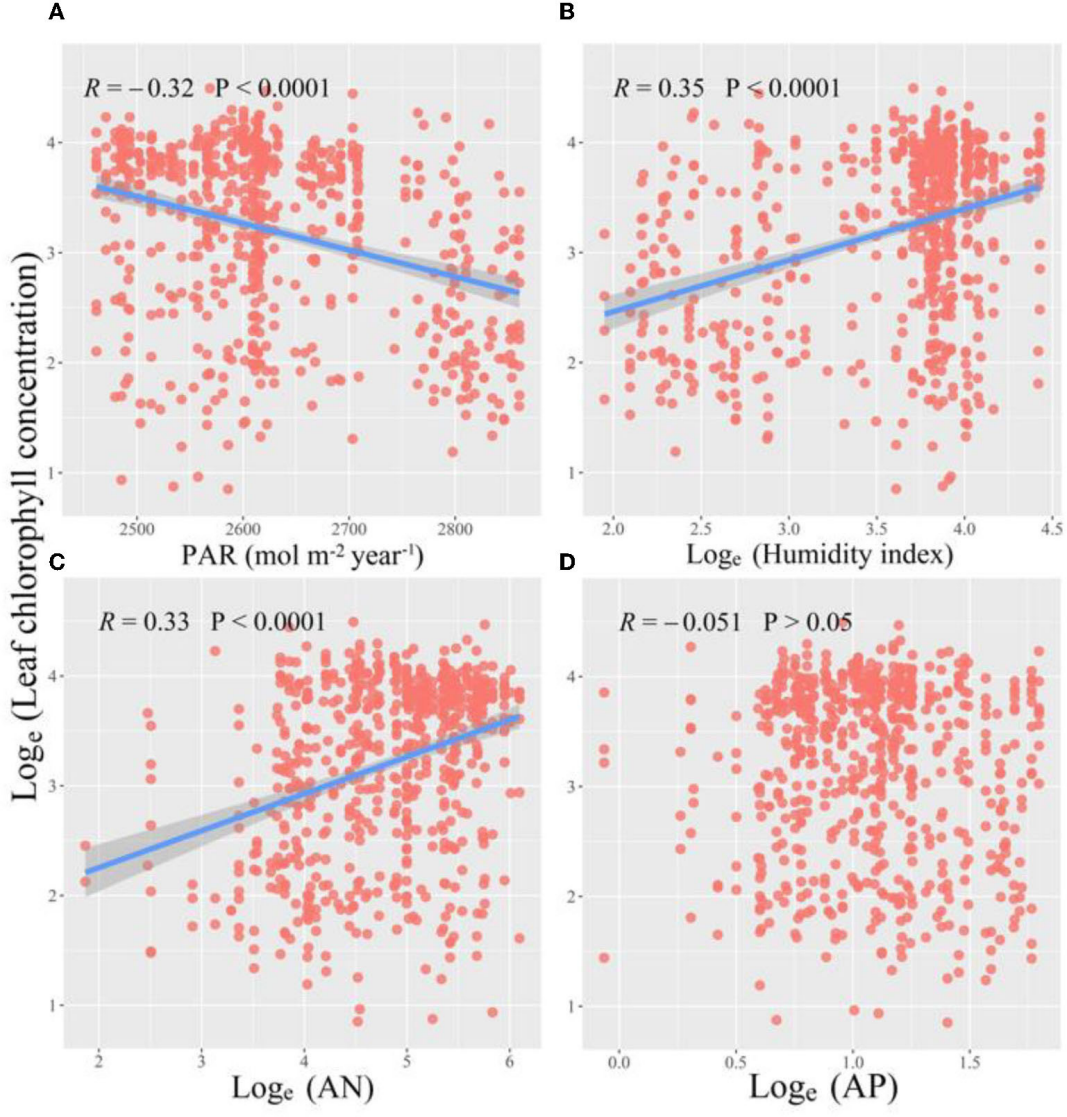
Relationships of leaf chlorophyll (Chl) with PAR [**(A)** photosynthetically active radiation], humidity index **(B)**, AN [**(C)** soil available nitrogen], and AP [**(D)** soil available phosphorus]. Red dots represent species-specific observations in each site. The blue lines and shades represent the regression lines with a 95% confidence band.

Across grasslands on the Tibetan Plateau, a significant spatial pattern of leaf Chl was detected among 677 site species. Leaf Chl was lower with increasing latitude or decreasing longitude (Figures 1A,B), but showed no significant trend with altitude (Figure 1C). Along with environmental gradients, leaf Chl reduced with PAR significantly (Figure 2A), but increased with HI (Figure 2B). Furthermore, leaf Chl was enhanced by higher soil N availability (Figure 2C), but had no response to soil P availability (Figure 2D).

**Figure 3:**
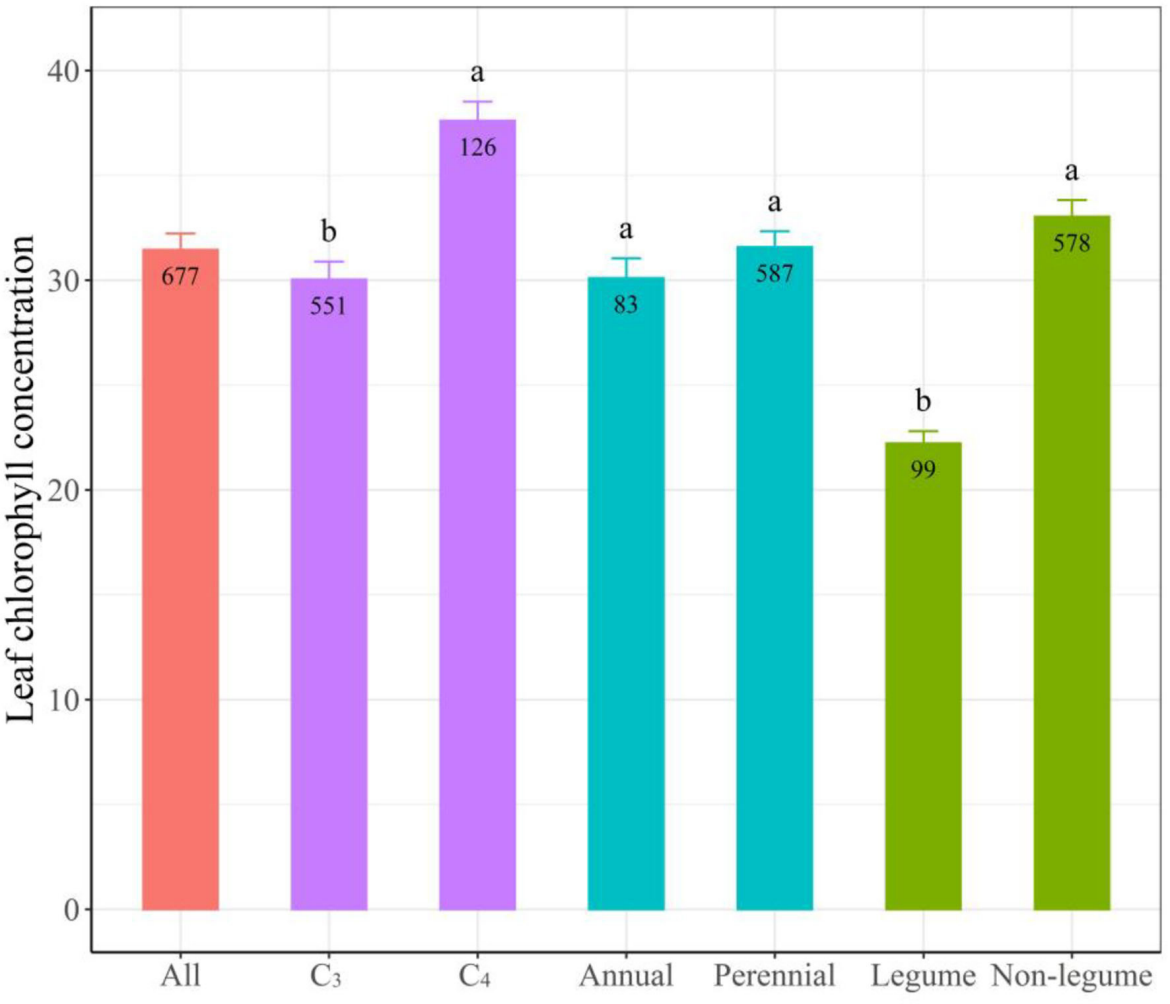
Leaf chlorophyll (Chl) in different functional groups. Different colors represent different comparisons of functional groups, such as C_3_ vs. C_4_ species, annual *vs*. perennial species, or legume vs. non-legume species. Bar height indicates the mean of leaf Chl with its standard error. Different letters represent a significant difference among different functional groups. The numbers in the bar represent the observations of leaf Chl in different functional groups.

Among different PFGs, we found a greater leaf Chl in C_4_ species (37.61 ± 0.91) than in C_3_ species (30.04 ± 0.85) (Figure 3). Leaf Chl in legume species (22.22 ± 0.59) was lower than in non-legume species (33.03 ± 0.79). However, leaf Chl showed no significant difference between annual and perennial species.

### The Dominant Role of Plant Evolution in Affecting Leaf Chl

**Figure 4:**
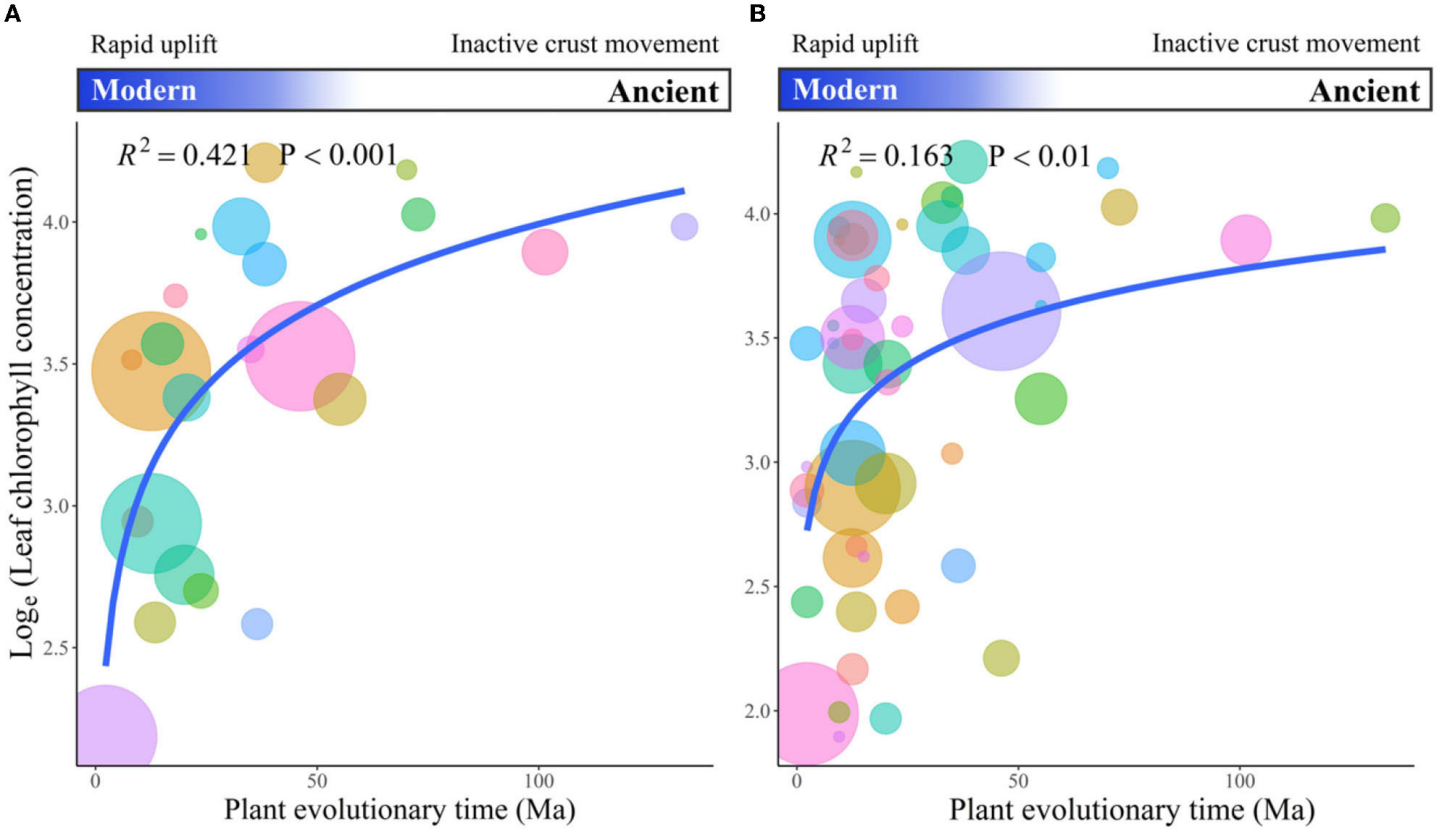
The relationships between leaf chlorophyll (Chl) and plant evolutionary time (million years) at both family **(A)** and genus levels **(B)**. Different colors represent different plant families or genera. The size of dots represents the number of site species at family or genus levels. The blue lines represent the regression lines.

Blomberg's *K* value indicated that the phylogenetic signal was not significant for leaf Chl among 93 species. Additionally, Pagel'λ values showed that variations in leaf Chl were mainly due to external environmental changes (not genetic variations) during plant evolutionary history (Supplementary Figure 3). Furthermore, we found that plant evolutionary divergence time was a good predictor for leaf Chl variations. A decreasingly evolutionary trend was detected for leaf Chl in a non-linear manner at both family (Figure 4A, *R*^2^ = 0.421, *p* < 0.001) and genus levels (Figure 4B, *R*^2^ = 0.163, *p* < 0.01). That is, modern species (lately evolved species) had a lower leaf Chl than ancestral species (early evolved species).

**Figure 5:**
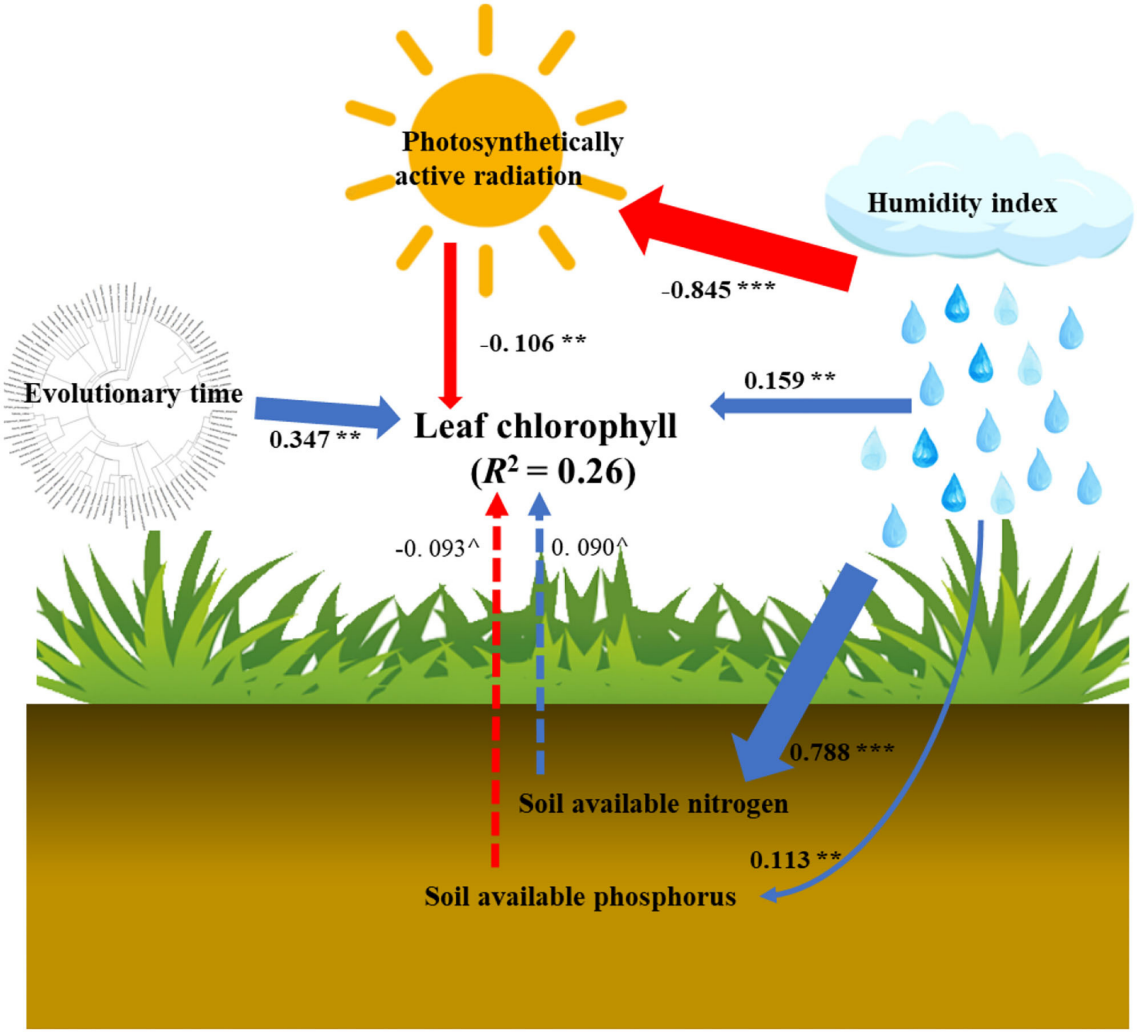
The direct and indirect impact pathways of plant evolutionary time, climate [humidity index, photosynthetically active radiation (PAR)], and soil nutrient [soil available nitrogen (N) and phosphorus (P)] in determining leaf Chl variations. The solid lines indicate significant pathways, while dashed ones represent marginally significant pathways (0.05 < *p* < 0.1). Blue lines are positive pathways, while red ones mean negative pathways. The numbers near the arrows represent standardized path coefficients, and the arrow width is proportional to the size of path coefficients. Significance levels are indicated by **p < 0.01; ***p < 0.001; ^∧^*p* < 0.1.

Based on the SEM analyses, we further quantified that plant evolution was the dominant driver for leaf Chl variations across the Tibetan Plateau (Figure 5). Specifically, the path coefficient of the plant evolution effect was 0.347. However, the sum of coefficients of other influencing pathways was lower than that of the plant evolution effect. The coefficient of the HI direct effect was 0.159. Those of the indirect effects of HI were 0.090 (HI→PAR→Chl), 0.071 (HI→soil N→Chl), and −0.011 (HI→soil P→Chl). The individual coefficients of PAR, soil available N and P were −0.106, 0.090, and −0.093, respectively.

## Discussion

As opposed to most previous studies conducted at the local scale (Croft et al., 2015, 2017; Lu et al., 2020), this study presented a large-scale pattern of leaf Chl among 677 site-species across grasslands on the Tibetan Plateau. Surprisingly, we found that plant evolutionary history was more dominant than current environmental changes in impacting the regional patterns of leaf Chl. This is different from the traditional view that leaf Chl is mainly affected by environmental factors, such as PAR (Genesio et al., 2020), soil water, and nutrient availability (Li et al., 2018b; Zhang et al., 2020). Moreover, we further detected the direction of evolutionary effects on leaf Chl, with the decreasing trend of leaf Chl along with evolution across the alpine grasslands. This Chl evolution response well-matches the increase in PAR during the uplift of the Tibetan Plateau. These indicate that the uplift of the Tibetan Plateau provides an ideal opportunity to study the evolutionary effects on plant traits. As leaf Chl is a fundamental component for photosynthesis, these results offer new insights into reconsidering plant photosynthesis capacity and the carbon cycle in an evolutionary view.

### The Spatial Patterns and Environmental Control of Leaf Chl

Across grasslands on the Tibetan Plateau, the spatial variations of leaf Chl concentration are mainly impacted by climate (PAR, HI), soil nutrients, and PFG. Along climate gradient, we revealed a decrease in leaf Chl with increasing PAR, which is consistent with our expectation and supported by previous studies (Niinemets, 2010; Genesio et al., 2020; Zhang et al., 2020). It is likely because plants are usually exposed to strong radiation across the Tibetan Plateau (Valladares and Niinemets, 2008). To avoid excessive radiation damage, alpine plants usually reduce leaf Chl and chloroplast number to offset the harmful effect of high radiation (photo-protection response), as well as to protect chloroplast interior structure (Klem et al., 2012; Genesio et al., 2020). Furthermore, alpine plants usually invest extra resources to protect them from suffering high radiation, such as cell membrane (thylakoid and mesophyll) thickening (Hallik et al., 2009). This perhaps leads to the reduction in resources for Chl biosynthesis and leaf Chl as well. Along the water gradient, leaf Chl was suppressed in drier environments (decreasing HI). This is caused by the fact that long-term drought stress could damage the thylakoid membrane and mesophyll cells, further reducing leaf Chl concentration (Alberte and Thornber, 1977). Besides, plants tend to invest more resources for survival under drought stress (Alberte and Thornber, 1977; Mihailović et al., 1997; Lei et al., 2006), indirectly decreasing resource allocation to leaf Chl (Bokhari, 1976; Estill et al., 1991; Mihailović et al., 1997).

Soil nutrient availability was also found to determine leaf Chl variations. The increase in leaf Chl with soil available N revealed in this study is in line with previous studies. For example, a positive relationship is normally detected between leaf N and Chl concentration (Croft et al., 2017; Zhang et al., 2020), and leaf N concentration is found to increase with soil available N (Zhao et al., 2003, 2005; Boussadia et al., 2011). This results from the fundamental role of the N element in constituting leaf Chl (Croft et al., 2017). In contrast, we found little effect of soil P availability on leaf Chl, likely caused by stronger N limitation than P limitation in alpine grasslands (Gao et al., 2018; Du et al., 2020).

A significant difference was detected among PFGs for leaf Chl as well. The greater leaf Chl in C_4_ than C_3_ species is partly due to the fact that C_4_ species have a stronger capacity to harvest CO_2_ than C_3_ species (Wang et al., 2011; Christin and Osborne, 2014). In contrast with our expectations and previous findings (Zhang et al., 2020), we revealed no difference in leaf Chl between annual and perennial species. Annual species are usually expected to invest more resources in fast aboveground growth and reproduction (high leaf Chl with resource-use strategy), but perennial species allocate more to belowground growth for the advantages in long-term persistence (low Chl with resource-conservative strategy) (Wright et al., 2004). However, across our grassland transect in the Tibetan Plateau, plants face multiple stresses of extremely low temperature, high radiation, and drought (Chen et al., 2013). Thus, the greatest pressure faced by alpine plants might be survival, not fast growth for resource competition. Furthermore, annual plants tend to invest more resources in reproduction. These together cause little difference in leaf Chl between annual and perennial species. Contrary to our expectation, we found a lower leaf Chl of legume than non-legume species. Generally, legumes have the advantage of obtaining soil N *via* rhizobium, possibly promoting leaf N and Chl (Zahran, 1999; Graham and Vance, 2003; Wei et al., 2019). Nevertheless, our results indicate that more N uptake in legumes in alpine may not be mainly used for leaf Chl biosynthesis. The symbiont of rhizobium costs a lot of resources and energy (Zahran, 1999). Besides, more N needs to be invested in resisting the stress of coldness, high radiation, and drought (Wang et al., 2018). These indicate that legume tends to increase investment in belowground resources and abiotic stress resistance, which may reduce aboveground allocation and further decrease leaf Chl. In addition, we found that leaf Chl in the grass species was lower than in the forb species (Supplementary Figure 5), which is supported by recent studies in alpine and temperate grasslands (Zhang et al., 2020).

### The Role of Plant Evolution in Affecting Leaf Chl

Across grasslands in the Tibetan Plateau, we revealed a decreasing trend of leaf Chl evolution among 677 site-species (93 species from 25 families). Our result is among the first to detect the direction of leaf Chl evolution, complementing previous studies that found the evolutionary divergence of leaf Chl among grassland species (Li et al., 2018a). This evolution pattern well-matches the gradual increase in PAR during the geological uplift of the Tibetan Plateau. Specifically, about 50 million years ago, the India-Eurasia continental collision rapidly uplifted the Tibetan Plateau until now (Royden et al., 2008). This geological uplift causes a gradual enhancement in PAR during plant evolutionary history (Blumthaler et al., 1997). Facing high radiation, plants do not necessarily invest more resources in leaf Chl for light capture (Qiu et al., 2011; Genesio et al., 2020; Zhang et al., 2020). To avoid strong radiation damage, alpine plants tend to evolve toward the strategy with lower leaf Chl. The additional resource from decreasing leaf Chl might be allocated to resist high radiation, such as densely villous aggregated globose, thicken bracts, and other morphological adaptions (Wen et al., 2014; Ibanez et al., 2017). Besides increased PAR, the temperature gradually reduces with the uplift (Zhisheng et al., 2001; Liu et al., 2006; Ge et al., 2014). On one hand, low temperature suppresses plant physiological activities (Bokhari, 1976; Fu et al., 2014; Li et al., 2018b), further reducing the biosynthesis of leaf Chl (Bokhari, 1976). On the other hand, low temperature forces alpine plants to invest more resources in cold resistance (Li et al., 2018b), likely cutting down allocation to leaf Chl during plant evolutionary history.

Moreover, we found that leaf Chl non-linearly declined with plant evolutionary divergence time, with a faster decrease recently. This non-linear evolution pattern also matches well with the different uplift stages. Before the continental collision (*c*. 50 million years ago), the crust movement is relatively inactive (Royden et al., 2008), and the evolutionary rate of leaf Chl was slow as revealed in our results. The Tibetan Plateau began to uplift after the collision (Rowley and Currie, 2006), while the uplift has been again accelerated about 20 million years ago (Royden et al., 2008; Wang et al., 2008). The different rates of the Tibetan Plateau uplift among multiple stages again support our results that the decreasing rates of leaf Chl evolution were quicker. These suggest that the uplift of the Tibetan Plateau provides an excellent opportunity for future studies to test the evolutionary effects on plant traits and functions. The greatest decrease in leaf Chl evolution recently calls our attention to the evolutionary constraint on plant photosynthesis and carbon cycle, especially for the regions where climate is quickly becoming warmer and wetter over the past decades (Chen et al., 2013).

Interestingly, the evolution of leaf Chl showed great variations in the lately evolved stage. This implies that some species evolve quickly toward the strategy with lower leaf Chl (Kita et al., 2004; Wang et al., 2004; Liu et al., 2013), but other species tend to inherit from ancestral traits when facing great environmental changes during evolutionary history (Wen et al., 2014; Volis et al., 2018). Furthermore, we found that the quickly evolved species were mainly coming from *Poaceae*, while the conservatively evolved species were from *Asteraceae* and C_4_ functional group. These suggest that it is interesting and valuable to study the mechanisms underlying different Chl evolutionary responses among different species with the uplift of the Tibetan Plateau, as well as the consequence for plant photosynthesis and the carbon cycle in alpine grasslands.

Overall, the SEM analysis concluded that plant evolution was the dominant driver for leaf Chl variations when compared with the individual or sum effects of current environmental changes (i.e., HI, PAR, and soil nutrient availability). Contrary to the traditional view that leaf Chl is mainly affected by climate, soil nutrients, and PFG (Zhang et al., 2020), our result highlights the dominant role of plant evolution in driving leaf Chl of alpine plants across the Tibetan Plateau. Interestingly, future efforts are deserved to quantify the other sources of great variations in leaf Chl across contrasting environments, such as soil Mg and Fe availability. As leaf Chl is fundamental to harvesting light for plant photosynthesis, these results have important implications as follows. (1) The geological uplift of the Tibetan Plateau offers an ideal opportunity to explore the impacts of plant evolution on leaf photosynthetic traits and the underlying mechanisms. (2) These results suggest the potential role of plant evolutionary history in photosynthetic capacity, which is largely ignored in previous studies. (3) Plant evolution might function on the ecosystem-level carbon cycle, arousing our awareness to reconsider the carbon budget across the Tibetan Plateau in an evolutionary view.

## Conclusion

As opposed to previously local-scale studies, our study presented a large-scale pattern of leaf Chl among 677 site-species across grasslands in the Tibetan Plateau. More importantly, we found that it was plant evolution, rather than current environment changes, dominantly shaping leaf Chl variations in alpine plants across contrasting environments. This challenges the traditional view that leaf Chl is mainly regulated by climate, soil resource, and PFG. Interestingly, we also detected the direction of evolutionary effects on leaf Chl, with a non-linear decreasing trend during plant evolution history. This evolutionary response well matches the non-linear enhancement in PAR during different uplift stages of the Tibetan Plateau. These suggest that the geological uplift of the Tibetan Plateau provides a great chance to study the impact of plant evolution on alpine plant traits and functions. These new findings arouse our attention to reconsidering ecosystem carbon balance across the Tibetan Plateau from an evolutionary perspective.

## Data Availability Statement

The original contributions presented in the study are included in the article/Supplementary Material, further inquiries can be directed to the corresponding author/s.

## Author Contributions

YH and DT designed the experiments. YH, RZ, and YL performed the field investigation and collected the data. YH, TL, and DT analyzed the data and wrote the paper. TL, DT, XC, and JL offered helpful comments. All authors contributed to the draft and revision of the manuscript.

## Funding

This study was financially supported by the Second Tibetan Plateau Scientific Expedition and Research (STEP) Program (Grant No. 2019QZKK0302), the National Key R&D Program of China (2017YFA0604802), the Kezhen-Bingwei Young Talents (2020RC003), and the Youth Innovation Promotion Association CAS (2021050).

## Conflict of Interest

The authors declare that the research was conducted in the absence of any commercial or financial relationships that could be construed as a potential conflict of interest.

## Publisher's Note

All claims expressed in this article are solely those of the authors and do not necessarily represent those of their affiliated organizations, or those of the publisher, the editors and the reviewers. Any product that may be evaluated in this article, or claim that may be made by its manufacturer, is not guaranteed or endorsed by the publisher.
