# Supplementary material for: Plant Evolution History Overwhelms Current Environment Gradients in Affecting Leaf Chlorophyll Across the Tibetan Plateau
Source: Front Plant Sci. 2022 Jul 11;13:941983. doi: 10.3389/fpls.2022.941983 (PMC9309890; doi:10.3389/fpls.2022.941983)
Supplement: Supplementary file 1 [file Data_Sheet_1.docx]

# Supporting Information

**Title:** Plant evolution history overwhelms current environment gradients in affecting leaf chlorophyll across the Tibetan Plateau

The following Supporting Information is available for this article:

**Fig. S1** Site locations across our grassland transect in the Tibetan Plateau**.** Black dots represent the sampling sites.

**Fig. S2** Relationships between humidity index and environment factors. Each point represents one site location.

**Fig. S3** The established phylogenetic tree for leaf chlorophyll among 93 species and the analyses of phylogenetic signals (Blomberg's K value and λ-value) across grasslands in the Tibetan Plateau.

**Fig. S4** The relationships between leaf Chl and plant evolutionary time (million years) at the species level. The blue lines and shades represent the regression lines with 95% confidence band.

**Fig. S5** Leaf Chl in forb and grass group. Different letters represent a significant difference in this group. The numbers in the bar represent the observations of leaf Chl.

**Table S1** Geographical located information and environmental conditions of our studied sites in the Tibetan Plateau.


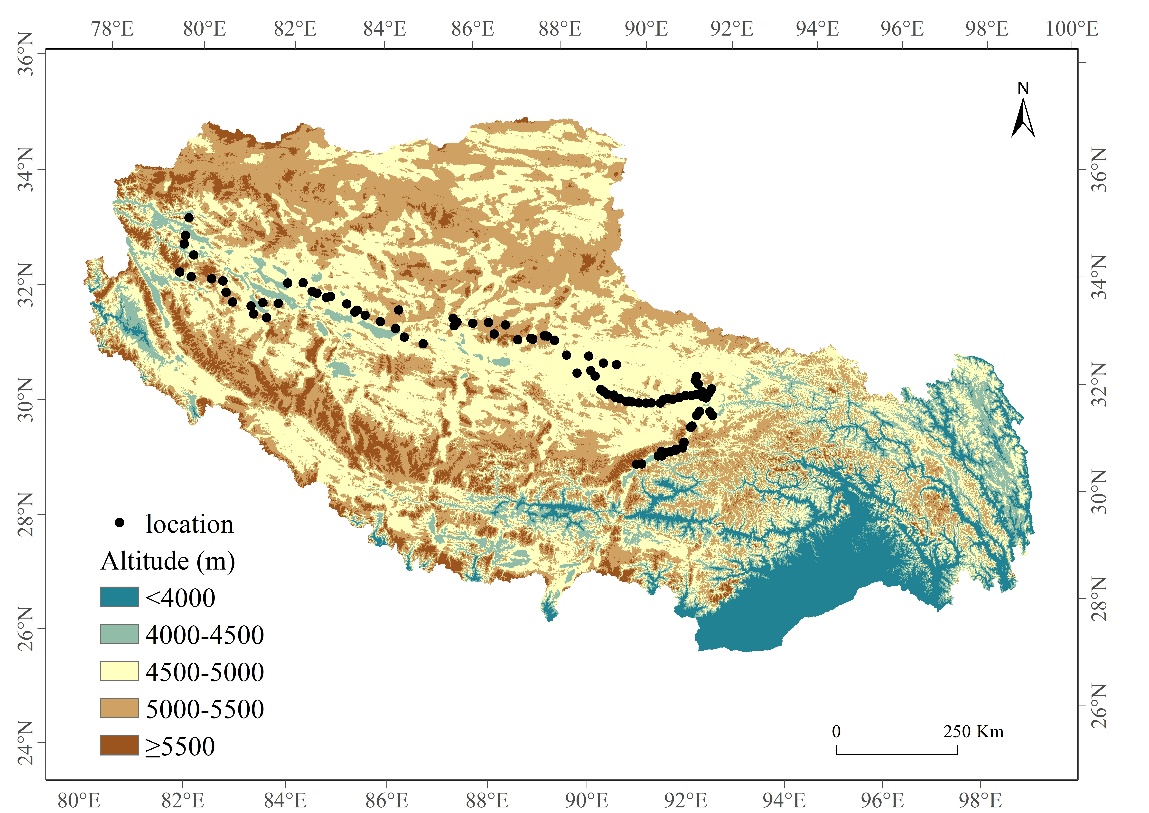


**Fig. S1** Site locations across our grassland transect in the Tibetan Plateau**.** Black dots represent the sampling sites.


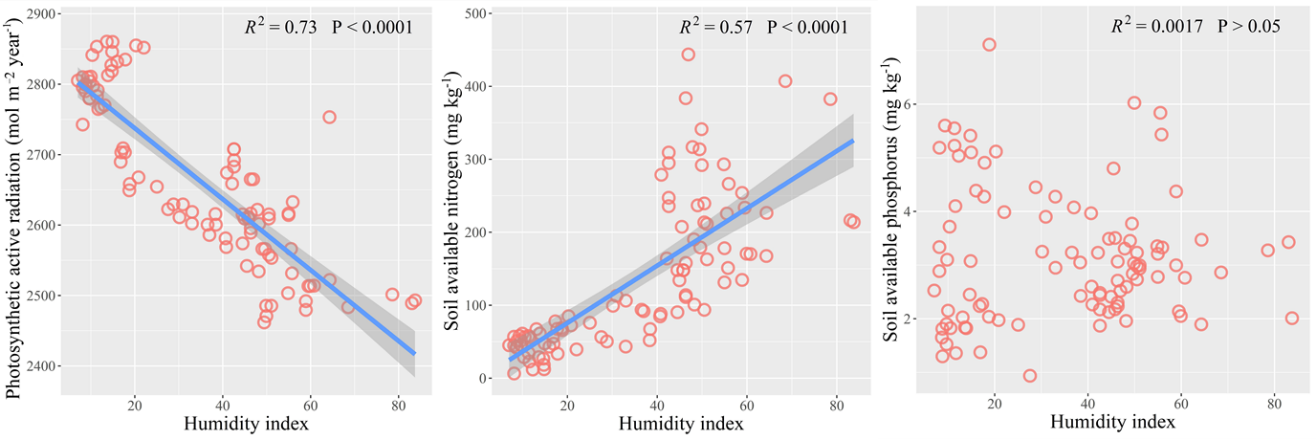


**Fig. S2** Relationships between humidity index and environment factors. Each point represents one site location.


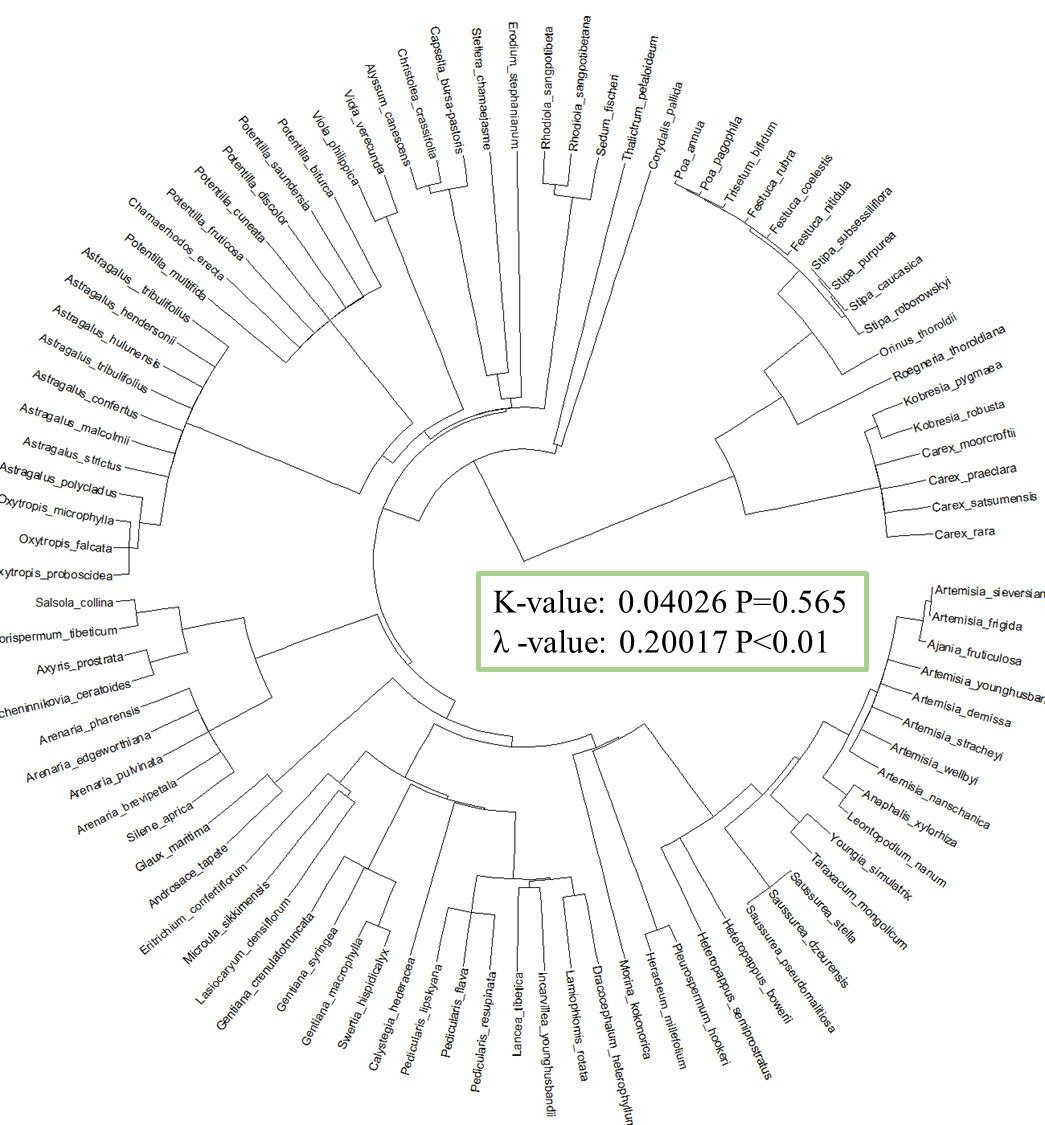


**Fig. S3** The established phylogenetic tree for leaf chlorophyll among 93 species and the analyses of phylogenetic signals (Blomberg's K value and λ-value) across grasslands in the Tibetan Plateau. The K-value of phylogenetic tree is 0.04026 (P=0.565) and λ-value is 0.20017 (*P*<0.01).


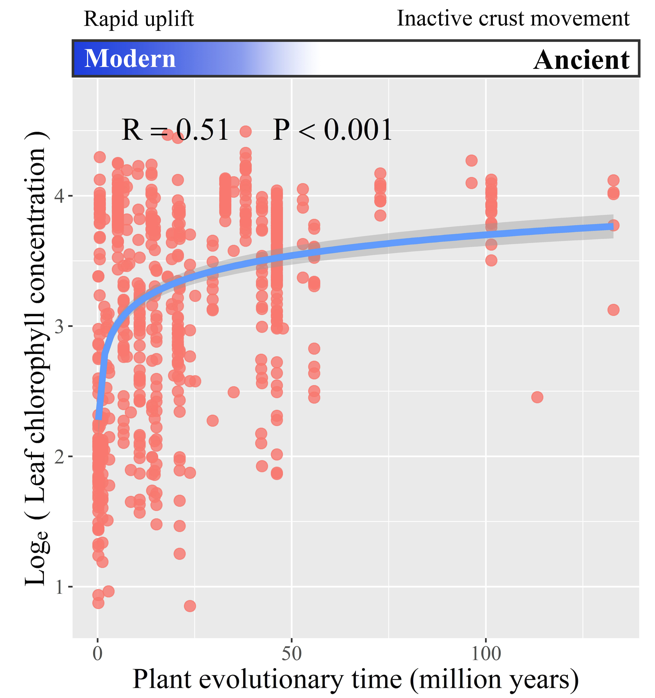


**Fig. S4** The relationships between leaf Chl and plant evolutionary time (million years) at the species level. The blue lines and shades represent the regression lines with 95% confidence band.


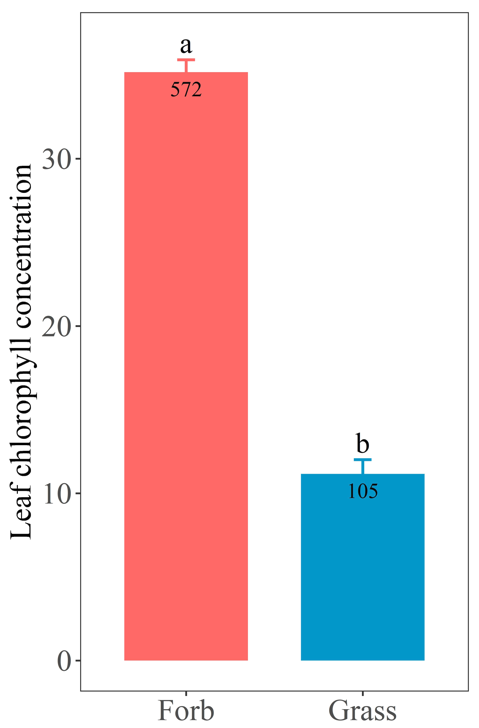


**Fig.** **S5** Leaf Chl in forb and grass group. Different letters represent a significant difference in this group. The numbers in the bar represent the observations of leaf Chl.

**Table S1** Geographical located information and environmental conditions of our studied sites in the Tibetan Plateau.

| Site | LAT (°N) | LONG (°E) | ALT (m) | PAR (mol·m^-2^·year^-1^) | MAP (mm) | MAT (℃) |
| --- | --- | --- | --- | --- | --- | --- |
| 1 | 33.34306 | 80.36944 | 4344 | 2766.921 | 61 | -5.03783 |
| 2 | 33.66833 | 80.3775 | 4389 | 2742.697 | 64 | -2.12948 |
| 3 | 33.18917 | 80.37917 | 4735 | 2783.133 | 79 | -3.05867 |
| 4 | 32.67556 | 80.39611 | 4461 | 2818.437 | 101 | -3.16102 |
| 5 | 33.02278 | 80.61944 | 4591 | 2791.361 | 89 | -2.29854 |
| 6 | 32.63167 | 80.6475 | 4542 | 2827.054 | 97 | -3.3766 |
| 7 | 32.65722 | 81.085 | 4772 | 2834.893 | 98 | -4.50288 |
| 8 | 32.65389 | 81.33111 | 4461 | 2831.887 | 96 | -3.9979 |
| 9 | 32.46083 | 81.4375 | 4578 | 2845.954 | 105 | -2.91838 |
| 10 | 32.30889 | 81.60694 | 4680 | 2851.903 | 102 | -5.37067 |
| 11 | 32.2925 | 82.01028 | 5016 | 2855.003 | 99 | -5.1141 |
| 12 | 32.15556 | 82.08944 | 4627 | 2860.447 | 105 | -2.29662 |
| 13 | 32.38528 | 82.24778 | 4575 | 2853.287 | 91 | -1.98971 |
| 14 | 32.12583 | 82.37583 | 4595 | 2860.043 | 114 | -2.36589 |
| 15 | 32.41694 | 82.57889 | 4465 | 2841.44 | 86 | -1.67036 |
| 16 | 32.80611 | 82.70167 | 4465 | 2812.637 | 81 | -4.16455 |
| 17 | 32.85778 | 83.03167 | 4386 | 2805.118 | 70 | -0.0395 |
| 18 | 32.72528 | 83.25139 | 4456 | 2810.735 | 74 | -2.58848 |
| 19 | 32.70694 | 83.35944 | 4464 | 2809.937 | 77 | -1.74256 |
| 20 | 32.65778 | 83.57361 | 4459 | 2810.033 | 77 | -0.52303 |
| 21 | 32.68778 | 83.66083 | 4613 | 2804.02 | 73 | -2.54681 |
| 22 | 32.59139 | 84.02444 | 4463 | 2795.743 | 75 | -0.78703 |
| 23 | 32.46528 | 84.22472 | 4481 | 2799.483 | 83 | -0.43699 |
| 24 | 32.50556 | 84.26806 | 4379 | 2797.635 | 80 | -2.4013 |
| 25 | 32.43333 | 84.455 | 4524 | 2799.353 | 83 | -0.5592 |
| 26 | 32.35694 | 84.79389 | 4483 | 2790.127 | 88 | 0.035475 |
| 27 | 32.26889 | 85.14056 | 4450 | 2780.17 | 96 | -0.07329 |
| 28 | 32.61306 | 85.15222 | 4606 | 2764.773 | 106 | -0.92385 |
| 29 | 32.13556 | 85.35139 | 4455 | 2779.627 | 96 | -0.07329 |
| 30 | 32.05944 | 85.77 | 4820 | 2770.101 | 108 | -1.76577 |
| 31 | 32.58528 | 86.34444 | 4922 | 2703.263 | 123 | -3.064 |
| 32 | 32.45333 | 86.3825 | 4780 | 2708.99 | 138 | -2.0311 |
| 33 | 32.52528 | 86.43889 | 4742 | 2703.263 | 146 | -1.37345 |
| 34 | 32.53944 | 86.77861 | 4713 | 2689.71 | 158 | -0.58073 |
| 35 | 32.58694 | 87.11972 | 4554 | 2658.303 | 184 | -0.24031 |
| 36 | 32.39 | 87.26667 | 4663 | 2667.827 | 229 | 0.992942 |
| 37 | 32.57611 | 87.48972 | 4536 | 2649.317 | 199 | 0.6134 |
| 38 | 32.33028 | 87.78861 | 4545 | 2654.403 | 246 | -0.1656 |
| 39 | 32.38472 | 88.06833 | 4510 | 2629.281 | 256 | -1.10763 |
| 40 | 32.3675 | 88.10889 | 4750 | 2629.281 | 317 | 0.243788 |
| 41 | 32.45333 | 88.35417 | 4650 | 2611.157 | 321 | 0.644662 |
| 42 | 32.45389 | 88.41889 | 4553 | 2622.497 | 293 | 0.630725 |
| 43 | 32.39194 | 88.58111 | 4591 | 2602.22 | 335 | 0.152079 |
| 44 | 32.13917 | 88.87444 | 4576 | 2618.687 | 362 | 0.959421 |
| 45 | 31.82639 | 89.13583 | 4565 | 2615.577 | 399 | 0.410008 |
| 46 | 32.15944 | 89.35222 | 4580 | 2585.963 | 375 | 0.141438 |
| 47 | 31.89333 | 89.42639 | 4590 | 2600.487 | 404 | 0.511092 |
| 48 | 31.80167 | 89.53028 | 4563 | 2600.58 | 390 | 0.671787 |
| 49 | 31.56639 | 89.67944 | 4611 | 2614.185 | 433 | -2.12451 |
| 50 | 32.05333 | 89.68417 | 4649 | 2581.2 | 410 | 0.085379 |
| 51 | 31.52861 | 89.74889 | 4587 | 2616.353 | 433 | -2.12451 |
| 52 | 31.48361 | 89.82528 | 4633 | 2616.353 | 426 | -0.81417 |
| 53 | 31.48056 | 89.9625 | 4740 | 2615.07 | 425 | -0.4496 |
| 54 | 32.0425 | 89.97028 | 4563 | 2568.773 | 417 | 0.226292 |
| 55 | 31.43972 | 90.00361 | 4772 | 2615.07 | 424 | -1.60325 |
| 56 | 31.42861 | 90.09056 | 4666 | 2609.277 | 424 | -1.60325 |
| 57 | 31.38583 | 90.24167 | 4629 | 2611.497 | 429 | -0.63533 |
| 58 | 31.3925 | 90.3125 | 4625 | 2609.327 | 429 | -0.63533 |
| 59 | 31.38417 | 90.37028 | 4631 | 2609.327 | 430 | -0.43399 |
| 60 | 31.3725 | 90.52167 | 4567 | 2601.71 | 428 | -1.13512 |
| 61 | 30.24 | 90.57 | 4181 | 2753.17 | 369 | -4.26151 |
| 62 | 31.38028 | 90.67333 | 4601 | 2595.54 | 426 | -0.81417 |
| 63 | 30.24944 | 90.67917 | 4498 | 2658.727 | 384 | -0.88794 |
| 64 | 31.39139 | 90.78833 | 4545 | 2589.29 | 426 | -0.81417 |
| 65 | 31.40139 | 90.98306 | 4578 | 2573.89 | 425 | -0.4496 |
| 66 | 30.42111 | 91.02083 | 4185 | 2707.777 | 371 | -1.28323 |
| 67 | 31.46778 | 91.04528 | 4588 | 2557.633 | 424 | -1.60325 |
| 68 | 30.51111 | 91.07917 | 4411 | 2692.008 | 371 | -1.28437 |
| 69 | 30.42417 | 91.09694 | 4216 | 2707.777 | 371 | -1.28323 |
| 70 | 31.49444 | 91.12306 | 4653 | 2553.45 | 429 | -1.60325 |
| 71 | 30.49167 | 91.15722 | 4288 | 2682.807 | 371 | -1.28323 |
| 72 | 31.48917 | 91.2425 | 4744 | 2541.94 | 426 | -0.63533 |
| 73 | 30.51583 | 91.27306 | 4269 | 2674.253 | 386 | -0.57193 |
| 74 | 30.55 | 91.37722 | 4387 | 2665.04 | 397 | -1.43675 |
| 75 | 31.52889 | 91.38694 | 4612 | 2531.543 | 433 | -2.23535 |
| 76 | 30.56917 | 91.38917 | 4425 | 2665.04 | 402 | -1.43675 |
| 77 | 31.57194 | 91.50528 | 4562 | 2521.98 | 433 | -3.27042 |
| 78 | 30.60417 | 91.52889 | 4527 | 2632.69 | 402 | -2.8117 |
| 79 | 30.70778 | 91.54889 | 4480 | 2621.83 | 398 | -1.68606 |
| 80 | 31.58056 | 91.64083 | 4635 | 2513.923 | 409 | -3.27042 |
| 81 | 30.99278 | 91.67111 | 4725 | 2566.163 | 416 | -1.61313 |
| 82 | 30.99 | 91.67167 | 4588 | 2566.163 | 411 | -1.61313 |
| 83 | 31.01667 | 91.70333 | 4706 | 2566.163 | 422 | -2.39998 |
| 84 | 31.86806 | 91.70333 | 4723 | 2491.927 | 433 | -2.64911 |
| 85 | 31.94083 | 91.71972 | 4628 | 2479.567 | 433 | -2.64911 |
| 86 | 31.60722 | 91.75056 | 4543 | 2503.28 | 432 | -2.12451 |
| 87 | 31.80194 | 91.76722 | 4842 | 2483.387 | 441 | -3.56553 |
| 88 | 31.21667 | 91.78639 | 4550 | 2534.25 | 422 | -1.2416 |
| 89 | 31.29583 | 91.84333 | 4585 | 2513.489 | 426 | -2.83919 |
| 90 | 31.29778 | 91.84361 | 4538 | 2513.489 | 429 | -2.83919 |
| 91 | 31.67278 | 91.85917 | 4590 | 2489.06 | 433 | -4.78538 |
| 92 | 31.57444 | 91.87861 | 4526 | 2485.437 | 432 | -1.53904 |
| 93 | 31.55444 | 91.96194 | 4613 | 2485.437 | 422 | -1.53904 |
| 94 | 31.64278 | 92.015 | 4586 | 2470.833 | 425 | -1.48946 |
| 95 | 31.30222 | 92.06389 | 4455 | 2493.128 | 436 | -4.79502 |
| 96 | 31.72889 | 92.07139 | 4664 | 2461.783 | 425 | -1.40187 |
| 97 | 31.23611 | 92.12833 | 4550 | 2501.417 | 409 | -4.79502 |
